# Skin Rash could Predict the Response to EGFR Tyrosine Kinase Inhibitor and the Prognosis for Patients with Non-Small Cell Lung Cancer: A Systematic Review and Meta-Analysis

**DOI:** 10.1371/journal.pone.0055128

**Published:** 2013-01-30

**Authors:** Hong-bing Liu, Ying Wu, Tang-feng Lv, Yan-wen Yao, Yong-ying Xiao, Dong-mei Yuan, Yong Song

**Affiliations:** 1 Respiratory Department, Jinling Hospital, Nanjing University School of Medicine, Nanjing, China; 2 Department of Respiratory Medicine, Jiangsu Province Geriatric Hospital, Jiangsu Province Geriatric Institute, Nanjing, China; Kyushu University, Japan

## Abstract

**Background:**

The aim of this study was to assess the role of skin rash in predicting the efficacy of epidermal growth factor receptor tyrosine kinase inhibitors (EGFR-TKIs) and the prognosis of patients with non-small cell lung cancer (NSCLC).

**Method:**

We systematically searched for eligible articles investigating the association between rash and the efficacy of EGFR-TKIs and the prognosis of patients with NSCLC. The summary risk ratio (RR) and hazard ratio (HR) were calculated using meta-analysis.

**Results:**

We identified 33 eligible trials involving 6,798 patients. We used two different standards to group the patients [standard 1: rash vs. no rash, standard 2: rash (≥ stage 2) vs. rash (stage 0, 1)]. For standard 1, the objective response rate (ORR) and disease control rate (DCR) of the rash group were significantly higher than the no rash group [RR = 3.28; 95% CI: 2.41–4.47(corrected RR = 2.225, 95% CI: 1.658–2.986); RR = 1.96, 95% CI: 1.58–2.43]. The same results were observed for standard 2. For standards 1 and 2, the progression-free survival (PFS) (HR = 0.45, 95% CI: 0.37–0.53; HR = 0.57, 95% CI: 0.50–0.65) and overall survival (OS) (HR = 0.40, 95% CI: 0.28–0.52; HR = 0.53, 95% CI: 0.35–0.71) of the rash group were significantly longer than the control group, and the same results were observed in the subgroup analysis.

**Conclusions:**

skin rash after EGFR-TKI treatment may be an efficient clinical marker for predicting the response of patients with NSCLC to EGFR-TKIs. Furthermore, skin rash is also the prognostic factor of patients with NSCLC. Patients with skin rash have a longer PFS and OS.

## Introduction

The discovery of epidermal growth factor receptor tyrosine kinase inhibitors (EGFR-TKIs) was a milestone in the development of non-small cell lung cancer (NSCLC) treatment. EGFR-TKIs mainly included gefitinib and erlotinib. EGFR mutations have been demonstrated to predict the efficacy of EGFR-TKIs in NSCLC [1], [2], [3]. In NSCLCs with EGFR mutations, the gefitinib objective response rate (ORR) was 71.2%; however, the gefitinib ORR for NSCLCs with wild type EGFR was less than 10% [4]. Therefore, it is important to ascertain the EGFR genotype of patients to predict the EGFR-TKI efficiency, though it is sometimes difficult to know the EGFR genotype of patients for various reasons. Thus, it is necessary to find other clinical markers that predict the EGFR-TKI efficacy in NSCLC.

Compared with traditional chemotherapy, the adverse events of EGFR-TKIs are small and include skin rash, diarrhea, fatigue, nausea, and elevated transaminases. Some studies revealed that skin rash was the most commonly reported adverse event [5]; the most common manifestation was an inflammatory follicular rash in the face, limbs and trunk rashes were less frequent [6]. A rash may affect the patient quality of life, and it may even result in a reduction in the drug dose or its withdrawal. However, many studies confirmed that patients with a skin rash may have a better response to EGFR-TKIs and an even better prognosis [7], [8], [9], [10]. In particular, Wacker, B et al. analyzed two large phase III studies (i.e., National Cancer Institute of Canada Clinical Trials Group (NCIC CTG) Study BR.21 and NCIC CTG Study PA.3). The BR.21 study evaluated single-agent erlotinib compared with placebo in patients with stage IIIB/IV non-small cell lung cancer who had failed at least one prior chemotherapy regimen. The PA.3 study evaluated erlotinib compared with placebo given in combination with standard gemcitabine therapy for patient treatment. This study concluded that rash development maybe a positive event that is indicative of a greater likelihood for clinical benefit [7]. However, the PA.3 study did not evaluated single-agent erlotinib. To further and systematically evaluate associations between skin rash and the efficacy of EGFR-TKIs and the prognosis of patients with non-small cell lung cancer, we performed a systematic review and meta-analysis of 33 studies to evaluate the role of skin rash in predicting the efficacy and PFS and OS of patients with non-small lung cancer treated with EGFR-TKIs.

## Materials and Methods

### Search Strategy

We performed an internet search of PubMed, the Embase database, the Cochrane library, the American Society of Clinical Oncology (ASCO), the European Society for Medical Oncology (ESMO) and the World Conference of Lung Cancer (WCLC) using the following terms: (gefitinib or erlotinib) AND (rash or skin) AND lung cancer. The deadline for trial inclusion was June 2012. The language was limited to English. The reference lists of all retrieved articles and those of relevant review articles were also cross-referenced. Eligible studies were those that reported or evaluated the amount of complete response (CR)+ the partial response (PR), or the CR+PR+ stable disease (SD) patients according to the Response Evaluation Standard in Solid Tumors (RECIST), the hazard ratio (HR) with the corresponding 95% confidence interval (CI) comparing overall survival (OS), progression-free survival (PFS) or time-to-progression (TTP) stratified by development of skin rash for patients with NSCLC who received monotherapy including erlotinib or gefitinib. Moreover, we excluded rashes caused by other diseases. Studies examining EGFR-TKIs in combination with other agents, such as cytotoxic agents, were excluded from the meta-analysis. Case reports, studies reporting 10 or fewer patients, and the same or overlapping data from the same authors were also excluded.

### Data Extraction

Two reviewers (Hongbing Liu and Ying Wu) independently collected the following data from all eligible studies: first author, year of publication, ethnicity, therapy line, the EGFR-TKI used, total number of cases and controls, number of patients with ORR (CR+PR) or disease control rate (DCR) (CR+PR+SD), HR with a 95%CI comparing the OS, PFS or TTP stratified by skin rash. Disagreements between the two reviewers were resolved by consensus, which involved a third reviewer (Yong Song). According to the National Cancer Institute Common Toxicity Standard, some studies used the presence or absence of a rash to distinguish cases and controls (standard 1). In other studies, cases were defined as patients with a rash that was ≥ stage 2, and controls were patients with a rash that were ≤ stage 1 (standard 2). Additionally, three trials [7], [11], [12]provided the data in both the two standards. Thus, we extracted data according to the two standards.

### Statistical Analysis

For studies in which the HR was not given directly, Kaplan-Meier plots were used to calculate the HR according to the methods described by Tierney [13].The risk ratio (RR) was used for the ORR and DCR, and the HR was used for PFS and OS. A *P*<0.05 was considered statistically significant. An RR >1 reflected a better overall response rate in the experimental arm. Begg tests were performed to examine whether there was a publication bias.

Analysis was performed using the STATA SE 12.0 package (StataCorp, College Station, TX). If the *P* value of heterogeneity assessment was found to be <0.05, the assumption of homogeneity was deemed in-valid and the random-effects model was used. Otherwise, the fixed-effects model was used. *P* values for all comparisons were two-tailed and statistical significance was defined as a *P*<0.05.

### Ethics and Funding Source

This was a literature-based study, and ethics approval was not required.

## Results

### Study Identification

**Figure 1:**
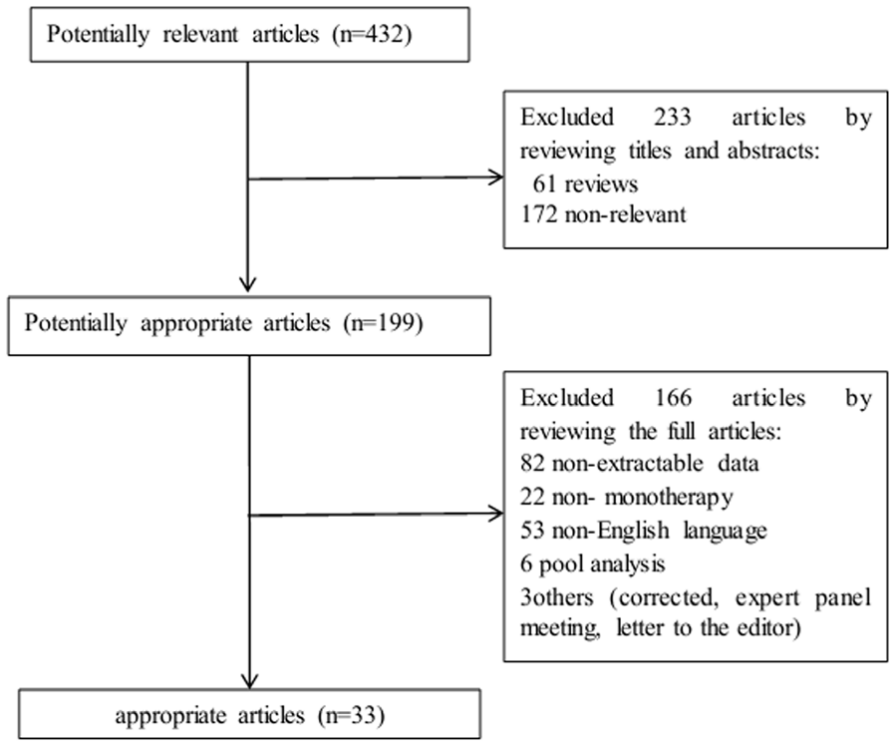
Flow chart demonstrating the progression of the trials in the review. The flowchart of selecting procedure and the exclusive reason of studies are summarized.

As shown in the NSCLC flow chart (Figure 1), our initial search yielded 432 potentially relevant published articles. A review of the titles and abstracts of these articles resulted in 199 promising articles. The remaining 199 articles were selected for analysis and evaluated in greater detail by reviewing the full articles. Of these, 166 articles were excluded for various reasons. Finally, 33 studies [5], [7], [8], [9], [10], [11], [12], [14], [15], [16], [17], [18], [19], [20], [21], [22], [23], [24], [25], [26], [27], [28], [29], [30], [31], [32], [33], [34], [35], [36], [37], [38], [39] with 6,798 patients were included in the meta-analysis. The characteristics of the eligible studies are summarized in Table 1.

**Table 1:** Characteristics of NSCLC Studies of EGFR-TKIs Included in Analyses.

							ORR(CR+PR)		DCR(CR+PR+SD)		PFS	OS
Author	Year	Ethnicity	Therapy line	EGFR-TKIs	patients	Case/control	Case	Control	Case	Control	HR(95%CI)	HR(95%CI)
Mita, A. C. [8]	2011	White	> = 2	E	42	24/18	5/24	0/18	15/24	9/18	NR	NR
Tiseo, M. [18]	2010	White	> = 2	G	91	30/61	10/30	7/61	NR	NR	NR	NR
Perez-Soler, R. [20]	2004	White	> = 2	E	57	43/14	7/43	0/14	28/43	7/14	NR	0.068(0.011–0.125)
Uhm, J. E. [17]	2009	Asian	mixed	E	120	93/27	26/93	3/27	61/93	7/27	0.52(0.32–0.9)	0.35(0.20–0.63)
Johnson, J. R. [25]	2005	White	> = 2	E	488	363/125	37/317	1/107	NR	NR	NR	0.65(0.52–0.81)
Zhu, Y. J. [14]	2010	Asian	mixed	E	79	48/31	18/48	5/31	44/48	15/31	0.258 (0.11–0.61)	NR
Mohamed,M. K. [22]	2005	White	> = 2	G	179	62/117	NR	NR	NR	NR	NR	0.53(0.39–0.73)
Park, J. [21]	2004	Asian	> = 2	G	111	67/44	26/67	3/44	36/67	8/44	NR	NR
West, H. L. [5]	2006	White	mixed	G	136	112/24	21/112	0/24	NR	NR	NR	0.45(0.27–0.76)
Liam, C. K. [23]	2006	Asian	mixed	G	23	14/9	9/14	2/9	NR	NR	NR	NR
Janne, P. A. [26]	2004	White	> = 2	G	154	73/81	5/73	2/81	40/73	27/81	NR	0.48(0.33–0.70)
Jackman, D. M. [27]	2007	White	mixed	E	80	63/17	NR	NR	NR	NR	NR	0.37(0.21–0.67)
Heigener, D. F. [28]	2011	White	mixed	E	2983	2299/684	NR	NR	NR	NR	0.52(0.48–0.57)	0.50(0.45–0.55)
Pircher, A. [10]	2011	White	mixed	G	74	35/39	6/35	2/39	23/35	9/39	0.419(0.214–0.82)	0.45(0.28–0.73)
Argiris, A. [33]	2006	White	mixed	G	39	15/24	NR	NR	9/15	5/24	NR	0.43(0.22–0.84)
Cadranel, J. [32]	2009	White	1	G	88	52/36	9/52	0/36	19/52	3/36	0.42(0.24–0.74)	0.30(0.17–0.54)
Chen, X. [30]	2011	Asian	1	G	61	33/28	NR	NR	NR	NR	0.39(0.19–0.59)	NR
Dudek, A. Z. [29]	2006	White	mixed	G	76	22/54	NR	NR	NR	NR	0.512(0.273–0.962)	NR
Veronese, M. L. [16]	2005	White	> = 2	G	101	48/53	10/48	1/53	NR	NR	NR	0.3(0.2–0.6)
Melosky B. [34]	2008	White	> = 2	E	35	41237	13/24	1/11	19/24	4/11	NR	NR
Chiu, C. H. [36]	2004	Asian	mixed	G	69	55/14	23/55	1/14	NR	NR	NR	NR
Lara-Guerra, H. [37]	2009	White	1	G	35	17/18	1/17	3/18	NR	NR	NR	NR
Mazzoni, F. [38]	2011	White	> = 2	E	53	27/26	NR	NR	12/27	8/26	NR	0.45(0.21–0.97)
Faehling, M. [39] *	2010	White	mixed	E	121	NR	NR	NR	NR	NR	0.46(0.29–0.72)	0.54(0.33–0.88)
Lilenbaum,R [35] *	2008	White	1	E	52	21(≥2)/31(<2)	NR	NR	NR	NR	0.45(0.25–0.81)	NR
Lee, Y [24] *	2012	Asian	> = 2	G+E	71	17(≥2)/54(<2)	3/17	3/52	NR	NR	0.54 (0.30–0.98)	0.59(0.30–1.13)
Platania, M. [19] *	2011	White	> = 2	E	43	14(≥2)/29(<2)	3/14	3/29	6/14	17/29	NR	NR
Hata, A. [9] *	2011	Asian	mixed	E	41	12(≥2)/29(<2)	NR	NR	10/12	8/29	0.41(0.21–0.81)	NR
Zhou, S. [15] *	2009	Asian	> = 2	E	112	52(≥2)/60(<2)	NR	NR	50/52	36/60	NR	NR
Cedres, S. [31] *	2009	White	> = 2	E	62	20(≥2)/42(<2)	2/20	4/42	9/20	13/42	0.57(0.33–0.97)	0.48(0.27–0.85)
Wacker, B. [7] †	2007	White	> = 2	E	389	197(2+)/115(1)/77(0)	38/312(1+);26/197(2+)	8/77(0);20/192 (0,1)	176/312(1+);118/197(2+)	12/77(0);70/192(0,1)	0.38(0.31–0.46)	0.33(0.26–0.39)
Perng, R. P. [11] †	2008	Asian	mixed	E	273	154(2+)/90(1)/29(0)	75/244(1+);55/154(2+)	3/29(0);23/119(0,1)	187/244(1+);126/154(2+)	12/29(0);73/119(0,1)	0.33(0.23–0.48); 0.57(0.44–0.73)	NR
Tiseo, M. [12] †	2009	White	mixed	E	460	198(2+)/134(1)/128(0)	NR	NR	227/332(1+);145/198(2+)	66/128(0);148/262(0,1)	0.7(0.57–0.85); 0.68(0.56–0.82)	NR

NOTE: The White ethnicity means the majority of patients in this study are White. Asian is also the same.

**Abbreviations:** NR: no report;

* The six studies with the grouping standard 2.

† The three studies patients were divided into three groups according to rash stage.

### Response Rate

**Figure 2:**
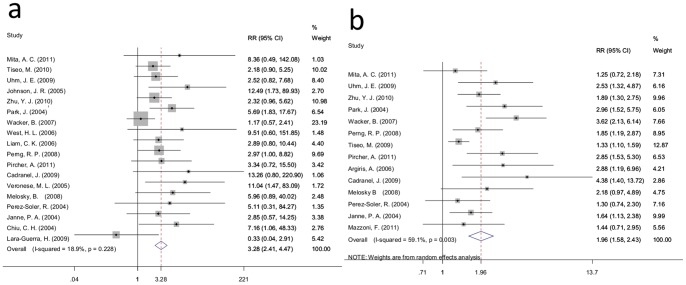
Forest plot of the RR for ORR and DCR for standard 1. a:ORR;b: DCR. The squares and horizontal lines correspond to the study-specific RR and 95% CI. The area of the squares reflects the weight (inverse of the variance). The diamond represents the summary RR and 95% CI.

For standard 1, data for the ORR and DCR was available for 18 and 14 trials, respectively. Analysis of these data demonstrated that the ORR of the rash group was 21.08% (339/1608), which was higher than the 6.06% (42/693) found for the no rash group (RR = 3.28; 95% CI: 2.41–4.47; I-squared = 18.9%, *P* = 0.228) (Figure 2a). While the overall DCR were 64.51% (896/1389) and 32.82% (192/585) for the rash and no rash groups, respectively. Meta-analysis revealed that the DCR of the rash group was nearly twice than that of the no rash group (RR = 1.96, 95% CI: 1.58–2.43; I-squared = 59.1%, *P* = 0.003) (Figure 2b).

Furthermore, subgroup analysis of therapy lines (i.e. ≥2 and mixed), ethnicity (i.e., White and Asian) and treatment (i.e., erlotinib and gefitinib) revealed that the ORR was significantly different between the two groups (therapy line: RR = 3.41, 95% CI: 2.24–5.20 and RR = 3.24, 95% CI: 1.99–5.29; ethnicity: RR = 3.20, 95% CI: 2.12–4.82 and RR = 3.39, 95% CI: 2.12–5.43; treatment: RR = 2.79, 95% CI: 1.84–4.22 and RR = 4.02, 95% CI: 2.52–6.40). The same results were also observed in the subgroup analysis for the DCR (therapy line: RR = 1.88, 95% CI: 1.34–2.64 and RR = 1.93, 95% CI: 1.44–2.58; ethnicity: RR = 1.90, 95% CI: 1.43–2.53 and RR = 2.08, 95% CI: 1.63–2.65; treatment: RR = 1.89, 95% CI: 1.46–2.46 and RR = 2.14, 95% CI: 1.46–3.13) (Table 2).

**Table 2:** Subgroup Meta-analysis of the ORR and DCR in Standard 1.

	ORR				heterogeneity		DCR				heterogeneity	
	studies	Rash	No rash	RR(95%CI)	I^2^	P	studies	Rash	No rash	RR(95%CI)	I^2^	P
Therapy line												
1	2	10/69	3/54	2.46(0.69–8.68)	78.50%	0.031	1	19/52	3/36	4.38(1.40–13.72)	–	–
≥2	9	151/938	23/466	3.41(2.24–5.20)	43.00%	0.081	7	326/570	75/271	1.88(1.34–2.64)	58.20%	0.026
mixed	7	178/601	16/173	3.24(1.99–5.29)	0.00%	0.916	6	551/767	114/278	1.93(1.44–2.58)	60.10%	0.028
Ethnicity												
White	12	162/1087	25/539	3.20(2.12–4.82)	39.60%	0.077	10	568/937	150/454	1.90(1.43–2.53)	65.50%	0.002
Asian	6	177/521	17/154	3.39(2.12–5.43)	0.00%	0.779	4	328/452	42/131	2.08(1.63–2.65)	0.00%	0.565
Treatment												
gefitinib	10	120/503	21/379	4.02(2.52–6.40)	11.40%	0.338	6	354/574	118/352	2.14(1.46–3.13)	69.30%	0.006
erlotinib	8	219/1105	21/314	2.79(1.84–4.22)	25.10%	0.229	8	542/815	74/233	1.89(1.46–2.46)	43.60%	0.088

**Figure 3:**
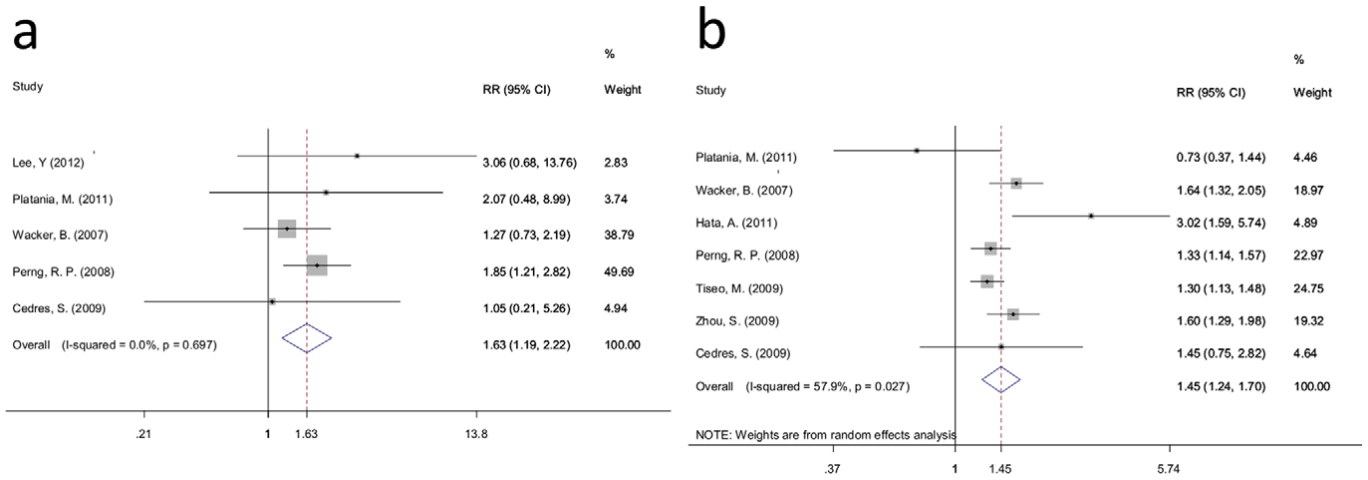
Forest plot of the RR for ORR and DCR for standard 2. a: ORR; b: DCR. The squares and horizontal lines correspond to the study-specific RR and 95% CI. The area of the squares reflects the weight (inverse of the variance). The diamond represents the summary RR and 95% CI.

For standard 2 studies, 5 trials reported ORR data, and 7 trials reported DCR data. The global ORR for the rash group (stage≥2) in the 5 trials was 22.14% (89/402), which was higher than the 12.21% (53/434) found for the control group (rash stage 0, 1) (RR = 1.63; 95% CI: 1.19–2.22; I-squared = 0.0%, P = 0.697) (Figure 3a). The overall DCR was 71.72% (464/647) for the rash group (stage≥2), and it was 49.80% (365/733) for the control group (rash stage 0, 1). Meta-analysis demonstrated that the DCR of the rash group (stage≥2) was higher that of the control group (rash stage 0, 1) (RR = 1.45, 95% CI: 1.24–1.70; I-squared = 57.9%, P = 0.027) (Figure 3b).

As demonstrated in Table 3, with the exception of the ORR for therapy lines ≥2, and White ethnicity had no significant difference between the two groups (RR = 1.41, 95% CI: 0.89–2.23; RR = 1.31, 95% CI: 0.80–2.13, respectively), the ORR for Asian, erlotinib and the DCR of the therapy line (i.e., ≥2 and mixed), ethnicity (i.e., White and Asian) between the two groups have a significant difference (RR = 1.91, 95% CI: 1.27–2.87; RR = 1.58, 95% CI: 1.15–2.18; RR = 1.49, 95% CI: 1.19–1.86; RR = 1.43, 95% CI: 1.14–1.79; RR = 1.35, 95% CI: 1.07–1.70; RR = 1.62, 95% CI: 1.22–2.15, respectively).

**Table 3:** Subgroup Meta-analysis of the ORR and DCR in Standard 2.

	ORR				heterogeneity		DCR				heterogeneity	
	studies	Rash(0,1)	Rash(≥2)	RR(95%CI)	I^2^	P	studies	Rash(0,1)	Rash(≥2)	RR(95%CI)	I^2^	P
Therapyline												
≥2	4	34/248	30/315	1.41(0.89–2.23)	0.00%	0.669	4	183/283	136/323	1.49(1.19–1.86)	41.70%	0.161
mixed	1	55/154	23/119	1.85(1.21–2.82)	−	−	3	281/364	229/410	1.43(1.14–1.79)	68.80%	0.041
Ethnicity												
White	3	31/231	27/263	1.31(0.80–2.13)	0.00%	0.794	4	278/429	248/525	1.35(1.07–1.70)	55.20%	0.082
Asian	2	58/171	26/171	1.91(1.27–2.87)	0.00%	0.527	3	186/218	117/208	1.62(1.22–2.15)	70.90%	0.032
Treatment												
gefitinib+erlotinib	1	3/17	3/52	3.06(0.68–13.76)	−	−	0	−	−	−	−	−
erlotinib	4	86/385	50/382	1.58(1.15–2.18)	0.00%	0.677	7	464/647	365/733	1.45(1.24–1.70)	57.90%	0.027

### Progression-Free Survival

**Figure 4:**
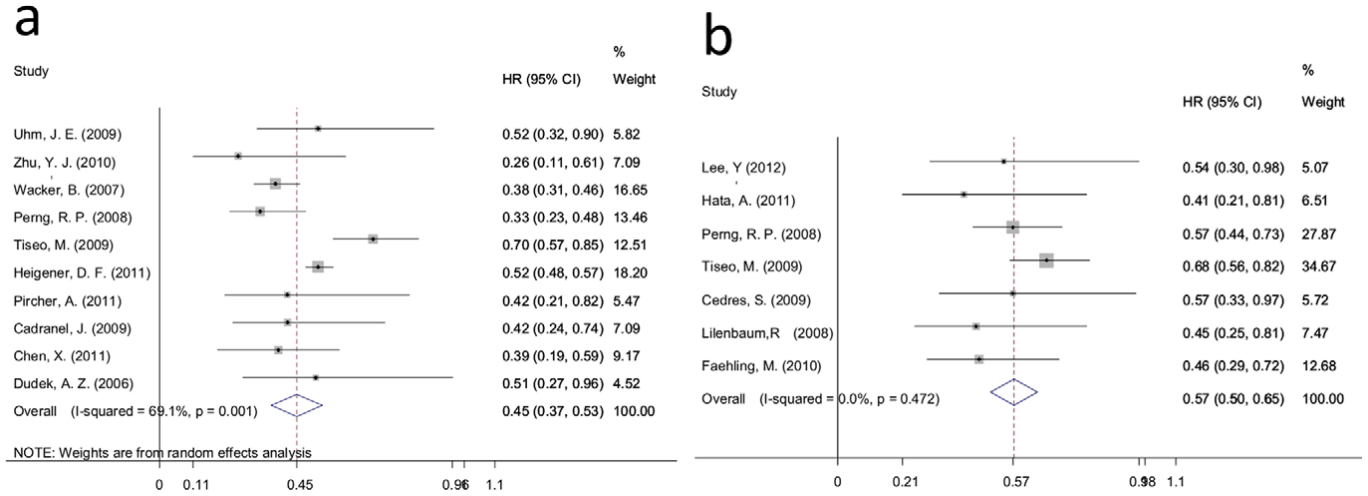
Forest plot of the HR for PFS for standard 1 and standard 2. a: standard 1; b: standard 2. The squares and horizontal lines correspond to the study-specific HR and 95% CI. The area of the squares reflects the weight (inverse of the variance). The diamond represents the summary HR and 95% CI.

For standard 1 studies, PFS data were available for 10 trials. Meta-analysis revealed that the risk of disease progression for patients with rashes decreased 55% compared with patients without a rash (HR = 0.45, 95% CI: 0.37–0.53; I-squared = 69.1%, *P* = 0.001) (Figure 4a). Further subgroup analysis of the therapy line (i.e., mixed and 1), ethnicity (i.e., Asian and White) and treatment (i.e., erlotinib and gefitinib) demonstrated that the risk of disease progression for patients with a rash decreased compared with patients without rash in every subgroup (therapy line: HR = 0.48, 95% CI: 0.36–0.59 and HR = 0.40, 95% CI: 0.25–0.56; ethnicity: HR = 0.35, 95% CI: 0.26–0.44 and HR = 0.50, 95% CI: 0.39–0.60; treatment: HR = 0.46, 95% CI: 0.35–0.57 and HR = 0.42, 95% CI: 0.29–0.55) (Table 4).

**Table 4:** Subgroup Meta-analysis of PFS.

	PFS(standard 1)		heterogeneity		PFS(standard 2)		heterogeneity	
	studies	HR(95%CI)	I^2^	P	studies	HR(95%CI)	I^2^	P
Therapy line								
1	2	0.40(0.25–0.56)	0.00%	0.854	1	0.45(0.17–0.73)	−	−
≥2	1	0.38(0.30–0.45)	−	−	2	0.56(0.32–0.79)	0.00%	0.900
Mixed	7	0.48(0.36–0.59)	69.20%	0.003	4	0.59(0.50–0.67)	36.10%	0.195
Ethnicity								
White	6	0.50(0.39–0.60)	73.80%	0.002	4	0.60(0.50–0.69)	28.70%	0.240
Asian	4	0.35(0.26–0.44)	0.00%	0.554	3	0.54(0.42–0.66)	0.00%	0.642
Treatment								
Gefitinib	4	0.42(0.29–0.55)	0.00%	0.948	0	−	−	−
Erlotinib	6	0.46(0.35–0.57)	82.10%	0.000	6	0.58(0.50–0.65)	9.70%	0.354
gefitinib+erlotinib	0	−	−	−	1	0.54(0.20–0.88)	−	−

For the standard 2 studies, PFS data were obtained for just 7 trials. Random effects analysis demonstrated that the risk of disease progression for patients with a rash (stage≥2) decreased 40% compared with patients with a rash (stage 0, 1) (HR = 0.57, 95% CI: 0.50–0.65; I-squared = 0.0%, *P* = 0.472) (Figure 4b). The risk of disease progression for patients with a rash (stage≥2) decreased compared with patients a rash (stage 0,1) in every subgroup in the therapy line (≥2 and mixed) ethnicity (Asian and White) and treatment (erlotinib)subgroup analyses (therapy line: HR = 0.56, 95% CI: 0.32–0.79 and HR = 0.59, 95% CI: 0.50–0.67; ethnicity: HR = 0.54, 95% CI: 0.42–0.66 and HR = 0.60, 95% CI: 0.50–0.69; treatment: HR = 0.58, 95% CI: 0.50–0.65) ) (Table 4).

### Overall Survival

**Figure 5:**
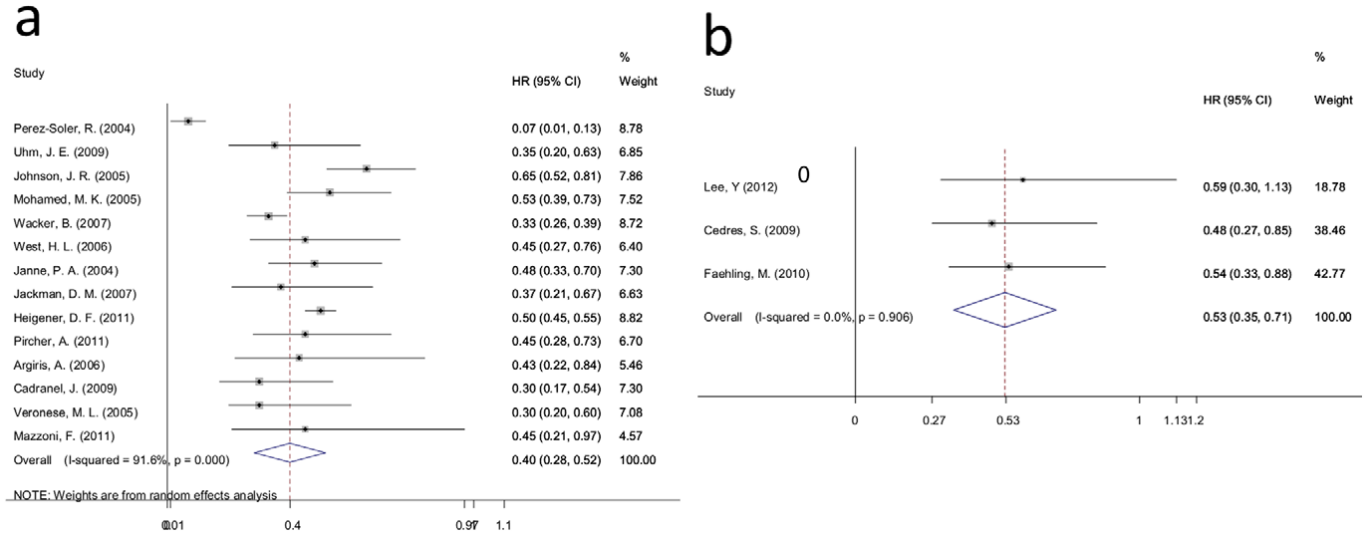
Forest plot of the HR for OS for standard 1 and standard 2. a: standard 1; b: standard 2. The squares and horizontal lines correspond to the study-specific HR and 95% CI. The area of the squares reflects the weight (inverse of the variance). The diamond represents the summary HR and 95% CI.

In the standard 1 studies, 14 trials reported OS data, and meta-analysis revealed that the risk of death for patients with a rash decreased 60% compared with patients without a rash (HR = 0.40, 95% CI: 0.28–0.52; I-squared = 91.6%, *P* = 0.000) (Figure. 5a).

Therapy line (≥2 and mixed), ethnicity (White) and treatment (erlotinib and gefitinib) subgroup analyses demonstrated that the risk of disease progression for patients with a rash decreased compared with patients without a rash in every subgroup (therapy line: HR = 0.39, 95% CI: 0.22–0.57 and HR = 0.48, 95% CI: 0.44–0.53; ethnicity: HR = 0.40, 95% CI: 0.28–0.53; treatment: HR = 0.38, 95% CI: 0.21–0.56 and HR = 0.42, 95% CI: 0.34–0.50) (Table 5).

**Table 5:** Subgroup Meta-analysis of OS in Standard 1.

	OS		heterogeneity	
	studies	HR(95%CI)	I^2^	P
Therapy line				
1	1	0.30(0.12–0.49)	−	−
≥2	7	0.39(0.22–0.57)	93.5%	0.000
mixed	6	0.48(0.44–0.53)	0.0%	0.684
Ethnicity				
White	13	0.40(0.28–0.53)	92.2%	0.000
Asian	1	0.35(0.13–0.56)	−	−
Treatment				
erlotinib	7	0.38(0.21–0.56)	95.9%	0.000
gefitinib	7	0.42(0.34–0.50)	0.0%	0.526

As shown in Figure 5b for the standard 2 studies, OS data were available for just 3 trials. Meta-analysis demonstrated that the risk of disease progression for patients with a rash (stage≥2) decreased 48% compared with patients with a rash (stage 0, 1) (HR = 0.53, 95% CI: 0.35–0.71; I-squared = 0.0%, *P* = 0.906).

### Publication Bias

**Figure 6:**
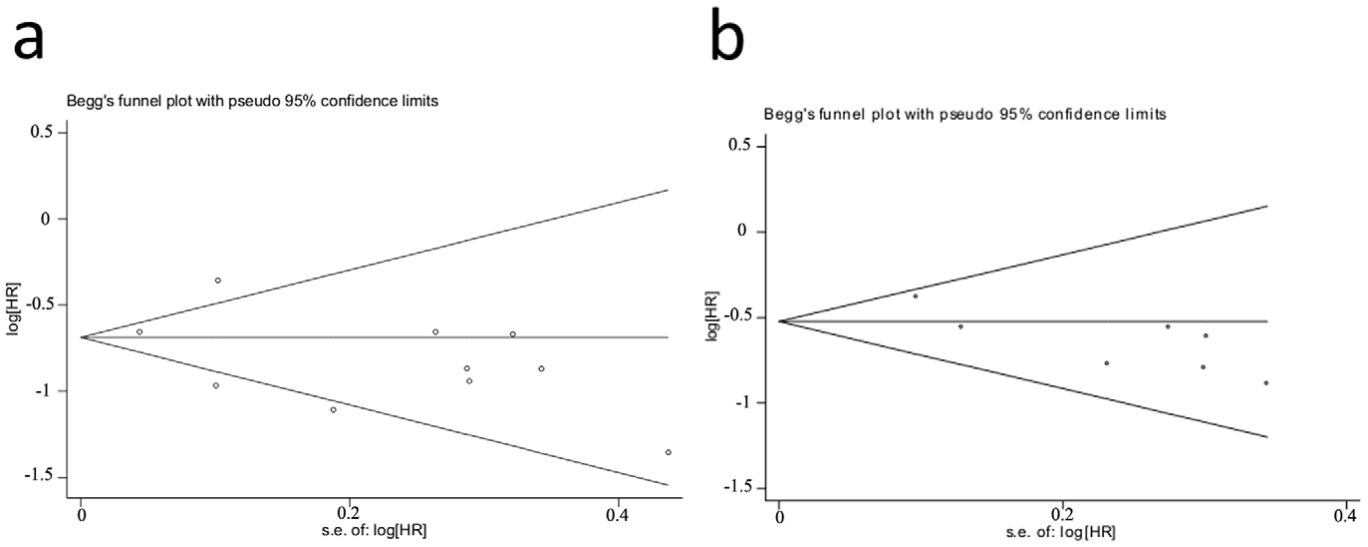
Funnel plot of the relative PFS ratios for standard 1 and standard 2. a: For standard 1 studies Begg test (*P* = 0.592). b: For the standard 2 studies with the Begg test (*P* = 0.368). Each point represents a separate study for the indicated association. Log [HR], natural logarithm of HR. Horizontal line, mean effect size.

To reduce publication bias, we conducted a more detailed literature search and experimental design. For standard 1, no publication bias analysis for DCR, PFS and OS was found according to funnel plot and Begg test (*P* = 0.189, *P* = 0.592 and *P* = 0.101) (Figure 6a). The same results were obtained for ORR, DCR, PFS and OS in standard 2 with the Begg test (*P* = 1.000, *P* = 0.764, *P* = 0.368 and *P* = 1.000) (Figure 6b). However, the publication bias was observed in the ORR analysis for standard 1 (*P* = 0.012). The method of trim and fill was used to correct the publication bias. The meta analysis showed that the corrected RR was 2.225 (95% CI: 1.658–2.986).

## Discussion

This study provides empirical evidence that skin rashes that occur after EGFR-TKI (i.e., gefitinib and erlotinib) treatment may be an efficient clinical marker for the prediction of the response of patients with NSCLC to EGFR-TKI treatment, including for the ORR and DCR. Furthermore, skin rashes are also associated with the PFS and OS of patients with NSCLC. Patients with skin rashes have a longer PFS and OS. The results of the therapy line, ethnicity (i.e., White and Asian) and treatment (i.e., gefitinib and erlotinib) subgroup analyses were similar. However, in the standard 2 subgroup analysis, there was no significant difference in the ORR of patients with ≥2 therapy lines or White ethnicity between the two groups. These results were not observed in the standard 1 subgroup analysis. Because the groupings in the included studies for skin rash are different, we used two different standards, standard 1 and standard 2, to group. Standard 1 compared rash vs. no rash, and standard 2 compared patients with stage 2 or greater rashes vs. those with a stage 0 or 1 rash. Thus, the difference in the subgroup analysis between standard 1 and 2 may be that the presence of a rash may be more efficient in predicting response than rash stage.

The presence of an EGFR gene mutation was used as a more efficient factor for predicting EGFR-TKI efficiency. The IPASS study revealed that the gefitinib response rate of NSCLC patients with EGFR mutations was approximately 70% [4]. However, in our study, the ORR of the rash group was only 21.10% (339/1607). This discrepancy may be explained as follows: First, most studies (24/33) included in this meta-analysis included a majority of white patients. As previously reported, White patients have a lower EGFR-TKI response rate than Asian patients. Second, most of the studies did not involve first-line EGFR-TKI treatment. In a study by Fausto Petrelli et al., the first-line EGFR-TKI response rate for patients with NSCLC was nearly 70%, but it was just 47.46% when used as a second-line or higher therapy [40]. Thus, the ORR of the rash group in this meta-analysis was lower.

We analyzed the data and found that rash incidence of patients with erlotinib was 76.54% and that of patients with gefitinb was 61.03%. So we conducted a subgroup analysis according to the two drugs.The subgroup analysis showed that the RR of gefitinb was correspondingly higher than erlotinib(Table 2). That means the relationship between the rash and efficiency for patients with gefitinib was stronger than that for patients with erlotinib. This might explained that the dose of erlotinib (150 mg) was the maximum tolerated dose (MTD), and the daily dose of gefitinib (250 mg) was only one-third of its MTD.

Skin rash is a main side effect of EGFR-TKI therapy and occur in approximately two-thirds of patients with NSCLC [41]. Skin toxicity is almost never lethal, but it may lead to the interruption or dose modification of anticancer agents [42], [43]. The mechanism of this side effect has not been fully elucidated. As is the case for cancer, the EGFR is important for the normal epidermis. EGFR is mainly expressed in undifferentiated, proliferating keratinocytes in the basal and suprabasal layers of the epidermis and the outer layers of the hair. EGFR-TKIs are thought to affect basal keratinocytes, leading to the development of skin rash side effects [41], [44]. Thus, skin rash in response to EGFR-TKI therapy may be an outward manifestation of the EGFR-TKI therapeutic effect on tumors, which may be explained by an association between skin rash and the EGFR-TKI efficiency even for the PFS and OS of patients from a molecular pathology perspective.

Because NSCLC prognosis is poor and the cost of EGFR-TKIs addition to the anticancer arsenal is substantial, it has become imperative that molecular or clinical markers are identified to stratify potential responders. This requirement has been highlighted in NSCLC with the identification of EGFR mutations correlating with responses to EGFR-TKIs and clinic pathological characteristics including sex, ethnicity, histology, and smoking history. Sometimes we cannot acquire genotypes of EGFR and other genes by reasons of tumor sample or technology unavailable in clinical. Therefore, we may need to select patients for EGFR-TKI treatment according to clinic pathological characteristics. Previous studies demonstrated that the EGFR-TKI response rate of White patients with NSCLC was lower than for Asians. However, our study revealed that patients with NSCLC that had a rash had a higher EGFR-TKI response rate compared with patients without a rash regardless of whether they were White or Asian. Thus, a skin rash may be more efficient in predicting the EGFR-TKI response rate of patients with NSCLC than clinic pathological characteristics. Furthermore, skin rash may be efficient prognostic factors for patients with NSCLC using EGFR-TKIs. The meta-analysis findings provide a useful basis for a clinician to judge the effectiveness of EGFR-TKI therapies for patients with NSCLC.

Although our meta-analysis revealed that skin rash was an efficient factor for predicting the response rate, PFS and OS of patients with NSCLC treated with an EGFR-TKI, skin rash can affect the patient quality of life, leading to dose reduction or even discontinuation, which may affect patient outcome. Although many drugs are used to treat skin rashes including topical skin moisturizers, topical sunscreens, and topical and systemic anti-inflammatory agents and antibiotics, they have not been clearly shown to be of clinical value [45], [46], [47]. Roman Perez-Soler et al. found that Menadione, at nontoxic concentrations, causes EGFR activation and may protect the skin from toxicity secondary to EGFR inhibitors without causing cytotoxicity [48].

Several cautions should to be taken into account when interpreting our results. First, a number of different factors may have affected the results including differences in the various studies, and the language limitation of the included studies, so the heterogeneity that exists in some of the meta-analyses. Second, the patient race in each study is often multiple, and we can only define patient race as the majority of the races accounted for in a study to the neglect the effect of a small number of races. Third, the number of first-line studies is small, and we cannot compare the different effects of rashes resulting from first- and second-line treatments. Forth, previous studies showed that it was approximately one month for occurrence of rash [20], [22], [49]. The OS of most patients included in this article was more than one month. However, there were still a few patients who did not live long enough for the occurrence of skin rash. Fifth, the publication bias was observed in the ORR analysis in standard 1(*P* = 0.012). We have searched as many databases and conference abstracts and could not find other related articles. Usually, it is easy to publish the positive results. And our study only included the published literature, so it may produce publication bias. Then the method of trim and fill was used to correct the publication bias. The Meta analysis showed that the corrected RR was 2.225 (95% CI: 1.658–2.986). So the skin rash was still associated with the ORR in standard 1. Despite the limitations of our study, we believe that it makes an important contribution to the NSCLC field.

In conclusion, we have reviewed the literature correlating skin rash, the efficacy of EGFR-TKIs, and the prognosis of patients with non-small cell lung cancer. Overall, the skin rash after using EGFR-TKI (i.e., gefitinib and erlotinib) may be an efficient clinical marker for predicting the response of patients with NSCLC to EGFR-TKIs including the ORR and DCR. Furthermore, skin rash was also associated with the PFS and OS of patients with NSCLC. Patients with a skin rash have a longer PFS and OS.
